# Co-assembly of graphene/polyoxometalate films for highly electrocatalytic and sensing hydroperoxide

**DOI:** 10.3389/fchem.2023.1199135

**Published:** 2023-05-18

**Authors:** Yayan Bao, Zezhong Chen, Yuzhen Wang, Lizhen Liu, Haiyan Wang, Zuopeng Li, Feng Feng

**Affiliations:** ^1^ School of Chemistry and Material Science, Shanxi Normal University, Linfen, China; ^2^ College of Chemistry and Environmental Engineering, Shanxi Datong University, Datong, China

**Keywords:** polyoxometalate, graphene, electrocatalytic, composite film, H_2_O_2_

## Abstract

Graphene oxide (GO) films mixed with polyethylenimine (PEI) were prepared by a layer-by-layer assembly (LBL) method, in which the GO component is then converted to reduced GO (rGO) *in situ* through an electron transfer interaction with a polyoxometalate (POM) that is assembled on the outer surface. With this, devices were manufactured by spreading composite films of (PEI/rGO)_n_-POM with different numbers of PEI/rGO layers on ITO substrates. Cyclic voltammetry (CV) reveals that the catalytic activity for H_2_O_2_ of (PEI/rGO)_n_-POM films was significantly higher than that of similar films of (PEI/GO)_n_/PEI/POM manufactured LBL with the same number of layers, although the catalyst POM content of (PEI/rGO)_n_-POM was only half that of (PEI/GO)_n_/PEI/POM. The catalytic activity of (PEI/rGO)_n_-POM films first increases and then decreases as the number of PEI/rGO layers increases. The result shows that (PEI/rGO)_3_-POM films with three PEI/rGO layers exhibit the highest efficiency. Amperometric measurements of the (PEI/rGO)_3_-POM films showed improved current response, high sensitivity, wide linear range, low detection limit, and fast response for H_2_O_2_ detection. The enhanced catalytic property of (PEI/rGO)_n_-POM films is attributed to the electron transfer interaction and electrostatic interaction between POM and rGO.

## 1 Introduction

Hydrogen peroxide (H_2_O_2_) is a powerful oxidizing agent, widely used to kill intestinal bacteria (Clifford and Repine, 1982), *Streptococcus* (Pericone et al., 2000), and purulent pathogenic yeasts (Levitz and Diamond, 1984), and for disinfecting surfaces (Barbut et al., 2009) However, high levels of H_2_O_2_ in humans would cause cell damage and have adverse health effects (Halliwell and Aruoma, 1991; Finkel and Holbrook, 2000). Therefore, several measures have been developed for the detection of H_2_O_2_ (Paolo et al., 1994; Feldman and Bosshart, 2002; Heng-Chia and Ho, 2015; Jiménez-Pérez et al., 2021). Among these techniques, electrochemistry (Zhang and Chen, 2017) is an important method for the detection of H_2_O_2_ due to its good stability, simple operation, and low detection limit. Designing a convenient and affordable H_2_O_2_ detector with high sensitivity, low detection limit, and fast response is the goal of current research efforts. Currently, new electrode-modifying materials are still being developed to meet the increasing and stringent demands.

Noble metal nanoparticles (Chen et al., 2011; Miao et al., 2014; Zhang et al., 2022) are the most commonly used materials for the preparation of modified electrodes. However, the high cost and complex preparation process of noble metal nanoparticles restrict their application. Transition metal oxides, such as Cu (Othmani et al., 2021), Mn (Yao et al., 2005), and Co (Zhang et al., 2016) oxides, are used as economically viable substitutes for noble metals, owing to their low price. However, the low stability of transition metal oxides in acidic media limits their application. Conductive polymers (Peng et al., 2009; Tao et al., 2014) have received much attention as mounting materials for electrochemical electrodes because of their convenient design and large film-forming area. However, the conductivity of conductive polymers is low, and increased use of conductive polymer materials will affect the conductivity and stability of the electrode (Liu et al., 2012).

Graphene, as a non-enzymatic electrode material for electrochemical detection of heavy metals (Xuan et al., 2016; Prasongpornet al., 2017), organic matter (Fan et al., 2012; Beitollahi et al., 2014), and reactive oxygen species (Xi et al., 2013), with electron transport capabilities and high surface area (Geim, 2009; Kauffman and Star, 2010), is the ideal nanomaterial for immobilized electrochemical catalysts. According to recent studies (Liu et al., 2012; Guo et al., 2013), the electrocatalytic activity displayed significant improvement if graphene was used as a catalyst support. Considering its cost-effectiveness, water compatibility, and ease to combine with other materials, graphene oxide (GO) is a suitable precursor for the large-scale production of graphene (Zhu et al., 2010; Chang and Wu, 2013). In addition, the oxygen-containing groups of GO can be reduced to reduced graphene oxide (rGO) by reducing catalysts, and the combination with rGO can improve the catalytic activity of catalysts. Furthermore, rGO films are a precondition for rGO-based catalytic devices due to the ease of separation. However, most rGO-based films were prepared by drop casting (Gilje et al., 2007; Du et al., 2015), layer-by-layer (LBL) (Yu et al., 2011; Hui et al., 2012), and electrodeposition methods (Liu et al., 2011a); these methods do not make it easy to obtain uniform rGO films or to combine directly with the catalyst. Not to mention that the catalytic efficiency of rGO-based films still needs to be improved. Thus, it is necessary to develop a method to overcome these obstacles.

Polyoxometalates (POMs) are a type of well-defined anionic cluster, consisting of transitional metal oxides (Müller et al., 1998; Yamase, 1998; Pope and Müller, 2001). POMs are suitable materials that act as a photocatalyst (Li et al., 2010; Li et al., 2011; Li et al., 2013; Li and Bubeck, 2013) or electrocatalyst (Li et al., 2013; Ahmed et al., 2022) for the reduction of GO to rGO due to their excellent redox properties. At the same time, POMs retain their structures and adsorb onto the rGO surface (Zhang et al., 2009), which could improve their catalytic activity. Therefore, we believe that rGO-POM films based on this method could greatly improve the catalytic activity of films.

Recently, we reported, for the first time, an example of *in situ* assisted electroreduction to manufacture rGO-based films with POM as a reducer and bridge molecules for a GO film and Au NPs; electrocatalysis for H_2_O_2_ (Zhang et al., 2019) and UA (Bao et al., 2020) showed that the film had enhanced catalytic activity. Compared to the alternately deposited LBL films of GO and POMs, films produced by *in situ* assisted electroreduction can greatly enhance the electrocatalytic activity due to rGO films having excellent electronic conductivity.

**SCHEME 1:**
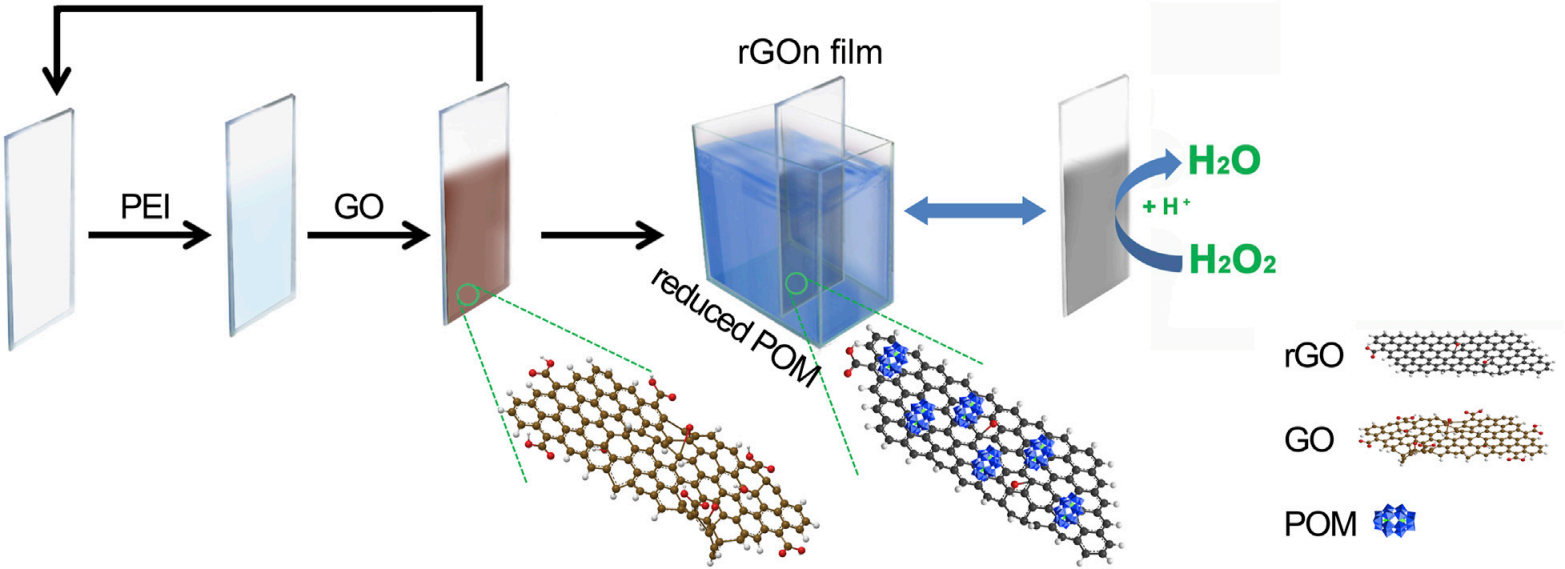
Schematic illustration of the fabrication procedure of (PEI/rGO)_n_-POM composite films by *in situ* assisted electroreduction.

Herein, we describe a new and easy approach to manufacture (PEI/rGO)_n_-POM films by reducing GO films (fabricated by LBL assembly) *in situ* with POM as the reducing agent, as shown in Scheme 1 . In this process, POM was adsorbed on rGO films by the electron transfer interaction that was beneficial for the electrocatalytic activity of the films. The catalytic activity for H_2_O_2_ of (PEI/rGO)_n_-POM films was much higher than that of similar films prepared by the LBL assembly with the same number of layers, even though the catalyst POM content was only half that of the latter. The (PEI/rGO)_3_-POM films exhibited remarkable improvement in electrocatalytic current response, sensitivity, and response time for H_2_O_2_ detection. In particular, the sensitivity of (PEI/rGO)_3_-POM films is the highest among similar films we recovered. Importantly, films based on rGO-POMs manufactured by *in situ* assisted electrochemical reduction can be treated as high-performance sensors for potentially catalytic activities.

## 2 Experimental

### 2.1 Materials

The polyoxometalate K_6_[P_2_W_18_O_62_]·19H_2_O (POM) was synthesized according to the literature (Richard et al., 1987) and identified by UV-Vis adsorption spectroscopy and cyclic voltammetry. Graphene oxide (GO) was synthesized according to the Hummers method mentioned in the literature (Li et al., 2011). Poly(ethylenimine) (PEI, MW 750,000) was purchased from Shanghai Macklin Biochemical Co., Ltd. Water purified using a Milli-Q–Milli-rho purification system with a resistivity of 18 MΩ cm was used in all experiments. All other chemicals of reagent grade were used as received.

### 2.2 Fabrication of composite films

Indium tin oxide (ITO) glass slides were cleaned before use according to the published procedures (Bi et al., 2008). The ITO substrates were cleaned by sonication in a 50% 1M NaOH in ethanol solvent for 5 min. Then, the ITO substrates were rinsed with deionized water for 1 min. The freshly prepared ITO substrates were immersed in a 1.0 wt% PEI solution (pH = 6.0) for 20 min, rinsed by dipping in deionized water twice for 1 min, and then cleaned by rinsing for 10 s. After that, the slide coated with PEI was immersed in a 0.3 mg/mL GO solution (pH = 7.0) for 20 min and rinsed as mentioned previously. The aforementioned steps were repeated, and the multilayer films were denoted (PEI/GO)_n_ where n is the number of layers of GO. Meanwhile, POM (10 mM, 10 mL) was reduced electrochemically at an applied potential of −1.0 V by electrolysis with vigorous stirring to improve the electrolysis rate. Bare ITO was used as the working electrode and a platinum wire and Ag/AgCl were used as counter and reference electrodes, respectively. After the solution turned dark blue (heteropoly blue), the substrate with (PEI/GO)_n_ films was carefully soaked in the solution with high-purity bubbled N_2_. Then, 20 min later, GO in the (PEI/GO)_n_ films was reduced by POM heteropoly blue, which resulted in rGO-POM hybrids. Films of (PEI/rGO)_n_-POM were formed. The fabrication procedure of the (PEI/rGO)_n_-POM composite films is schematically depicted in Scheme 1. For the purposes of comparison, the (PEI/GO)_n_/PEI/POM films were fabricated by the following steps. The substrate with (PEI/GO)_n_ films was dipped in a 1.0 wt% PEI solution for 20 min, followed by immersion in POM (10 mM, 10 mL) for 20 min. (PEI/GO)_n_/PEI/POM films were formed.

### 2.3 Characterization

UV-Vis absorption spectra were obtained using a Lambda 35 UV-Vis spectrometer with a slit width of 2 nm. Scanning electron microscopy (SEM) was performed with a Tescan MAIA-3 field emitting scanning electron microscope for surface morphology. Energy dispersive X-ray (EDX) was recorded using an Oxford X-act Energy Dispersive Spectrometer for compositional analysis of the composite films. X-ray photoelectron spectroscopy (XPS) was performed using an ESCALAB 250 Xi spectrometer with a monochromic X-ray source (Al Kɑ line, 1,486.6 eV).

### 2.4 Electrochemical experiments

Electrochemical experiments were performed with a CHI 660E electrochemical system. A standard three-electrode configuration was used. The composite films modified on the ITO electrode (ITO-coated glass slide) were used as the working electrode. A platinum wire and Ag/AgCl were used as counter and reference electrodes, respectively. The electrolyte used was a 0.5 M H_2_SO_4_–Na_2_SO_4_ buffer solution with a pH of 2.5. Before the electrochemical experiments, all the as-prepared composite films were pre-scanned by cyclic voltammetry (CV) to ensure that the curve was invariant. All experiments were carried out at room temperature.

## 3 Results and discussion

### 3.1 Assembly and structure of (PEI/rGO)_n_-POM

Here, we present the (PEI/GO)_n_ LBL films electroreduced *in situ* by using the POM cluster as an electrochemical reducer. In the LBL assembly process, GO nanosheets with a negatively charged surface (Liang et al., 2009) were linked by layers of cationic polyelectrolyte PEI through the electrostatic interaction. In the electroreduced *in situ* procedure, the (PEI/GO)_n_ films were soaked in the POM heteropoly blue solution by electroreduction at an applied potential of −1.0 V. POM acts as an electrocatalyst for GO in (PEI/GO)_n_ films. The dark blue color of the electroreduced POM originates from W^5+^ → W^6+^ intervalence charge transfer (Yamase, 1998), which could transfer electrons to GO in (PEI/GO)_n_ films. Then, GO was reduced to rGO while the electroreduced POM returned to its initial light-yellow state. At the same time, POM was adsorbed onto rGO as an anionic stabilizer (Li et al., 2010), as shown in Scheme 1. In this process, the surface of rGO can retain a small number of C–O groups, while the PEI layers with positive charge can still adsorb rGO with weak negative or neutral groups.

**FIGURE 1:**
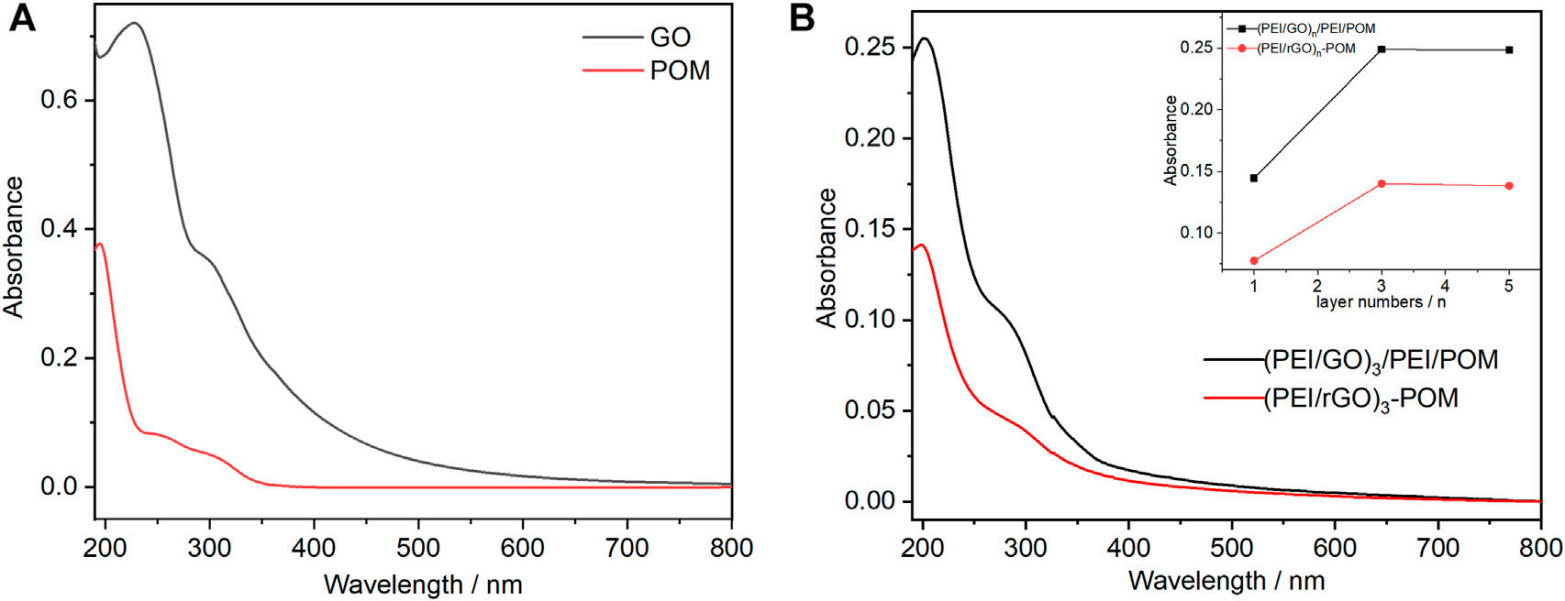
**(A)** UV-Vis absorption spectra of 0.001 mM POM aqueous solution (red line) and an aqueous 0.01 mg/mL GO solution (black line). **(B)** UV-Vis absorption spectra of (PEI/rGO)_3_-POM and (PEI/GO)_3_/PEI/POM films with different layers. Inset: plots of the absorbance values at 194 nm vs. the (PEI/GO) layer number n for (PEI/rGO)_n_-POM and (PEI/GO)_n_/PEI/POM films.

The UV-Vis characteristic absorptions of GO and POM are shown in Figure 1A. There are two absorption bands of GO appearing at 230 and 300 nm. The LMCT transition bands of POM are located at 194, 247, and 302 nm. The maximum wavelength of absorption was 194 nm. The extinction coefficient ε_194_ at 194 nm of POM can be calculated as 3.78 × 10^5^ M^−1^ cm^−1^ according to the absorption of POM with a concentration of 0.001 mM (Figure 1A). The POM content was easy to monitor for the (PEI/rGO)_n_-POM and (PEI/GO)_n_/PEI/POM films, according to the absorbance in the POM characteristic absorption bands. Figure 1B shows the UV-Vis absorption spectra of (PEI/rGO)_n_-POM and (PEI/GO)_n_/PEI/POM films (with *n* = 1, 3, and 5) assembled on quartz substrates. The UV-Vis absorption spectra of two types of n-layers composite films were observed based on (PEI/rGO)_n_ as reference films. Therefore, the absorbance originates from the POM content of the two types of composite films. Furthermore, the absorption peaks of the two types of films are similar to those of POM in solution rather than GO, which clearly shows that the UV-Vis absorption bands originate from POM. Interestingly, the UV-Vis absorption at 194 nm (belongs to POM) of (PEI/rGO)_n_-POM films is approximately half (to be exact 53%–57%) that of (PEI/GO)_n_/PEI/POM films (with the same GO layer numbers), regardless of the layer number (Inset of Figure 1B). Therefore, the POM content in (PEI/rGO)_n_-POM was only approximately half of that in (PEI/GO)_n_/PEI/POM films. This indicates that PEI sites in (PEI/GO)_n_/PEI/POM films appear to adsorb POM more than rGO sites in (PEI/rGO)_n_-POM films. Furthermore, comparing the absorbance in different layers of (PEI/rGO)_n_-POM films, it is clear that with an increasing number of layers, the POM content increases significantly first and then remains stable. This scenario can be attributed to the permeability of POM to (PEI/GO)_n_ films. An increased number of layers in the (PEI/rGO)_n_-POM films can carry more GO, which leads to more POM adsorption onto the GO surface in the manufacturing process. However, as the number of layers increases, the capacity of POM adsorbed by GO in (PEI/rGO)_n_-POM films does not increase due to blocking of POM mass transfer. A similar situation was observed in (PEI/GO)_n_/PEI/POM films as the POM content increases significantly first and then remains stable with increasing number of layers. This is probably due to an increase in the roughness of LBL films as the number of layers increases.

In terms of the ratio of Γ = (N_A_ A_λ_)/2ε_λ_ (N_A_ = 6.02 × 10^23^ mol^−1^), the surface coverage density Γ of the POM in each films can be calculated according to the UV-Vis spectra, where N_A_ is Avogadro’s constant, A_λ_ is the POM absorbance in each (PEI/rGO)_n_-POM or (PEI/GO)_n_/PEI/POM film with different layer numbers at a given wavelength λ, and ε_λ_ is the isotropic molar extinction coefficient of POM in λ (Xu et al., 2009). As ε_195_ was calculated to be 3.78 × 10^5^ M^−1^ cm^−1^ as mentioned previously, A_195_ was divided by 2 to obtain the absorbance for a single layer of POM. According to the abovementioned parameters, the average coverage density of the POM surface was obtained, as shown in Table 1. According to the crystalline structure of the POM, the average area per anion is 1.72 nm^2^ in the (001) plane, 2.56 nm^2^ in the (010) plane, and 3.33 nm^2^ in the (100) plane (Richard et al., 1987). Thus, the amount of POM surface coverage in all composite films is much greater than that of the crystalline material. This can be attributed to the large surface area of GO and the increase in surface roughness as the number of layers increases. However, compared to the (PEI/rGO)_1_-POM films, the average area per anion of the (PEI/rGO)_3_-POM and (PEI/rGO)_5_-POM films is smaller (Table 1). This could be caused by penetration of the POM into the GO inner layer. However, from (PEI/rGO)_3_-POM to (PEI/rGO)_5_-POM, the PEI/rGO layer number increased from three to five, which did not cause an increase in the average area per anion, indicating that the POM could not penetrate the deeper GO layers of the (PEI/rGO)_5_-POM. The issue of POM penetration is discussed in the XPS testing in the following section.

**TABLE 1:** UV-Vis absorbance (A_195_), surface coverage (anions/cm^2^), and area per anion [nm (Pericone et al., 2000)] for composite films.

Composite film	Absorbance	Surface coverage	Area per anion
(PEI/rGO)_1_-POM	0.078	6.21×10^13^	1.61
(PEI/GO)_1_/PEI/POM	0.145	11.5×10^13^	0.87
(PEI/rGO)_3_-POM	0.141	11.2×10^13^	0.89
(PEI/GO)_3_/PEI/POM	0.255	20.3×10^13^	0.49
(PEI/rGO)_5_-POM	0.136	10.8×10^13^	0.93
(PEI/GO)_5_/PEI/POM	0.243	19.4×10^13^	0.52

### 3.2 Structure characterization of composite films

**FIGURE 2:**
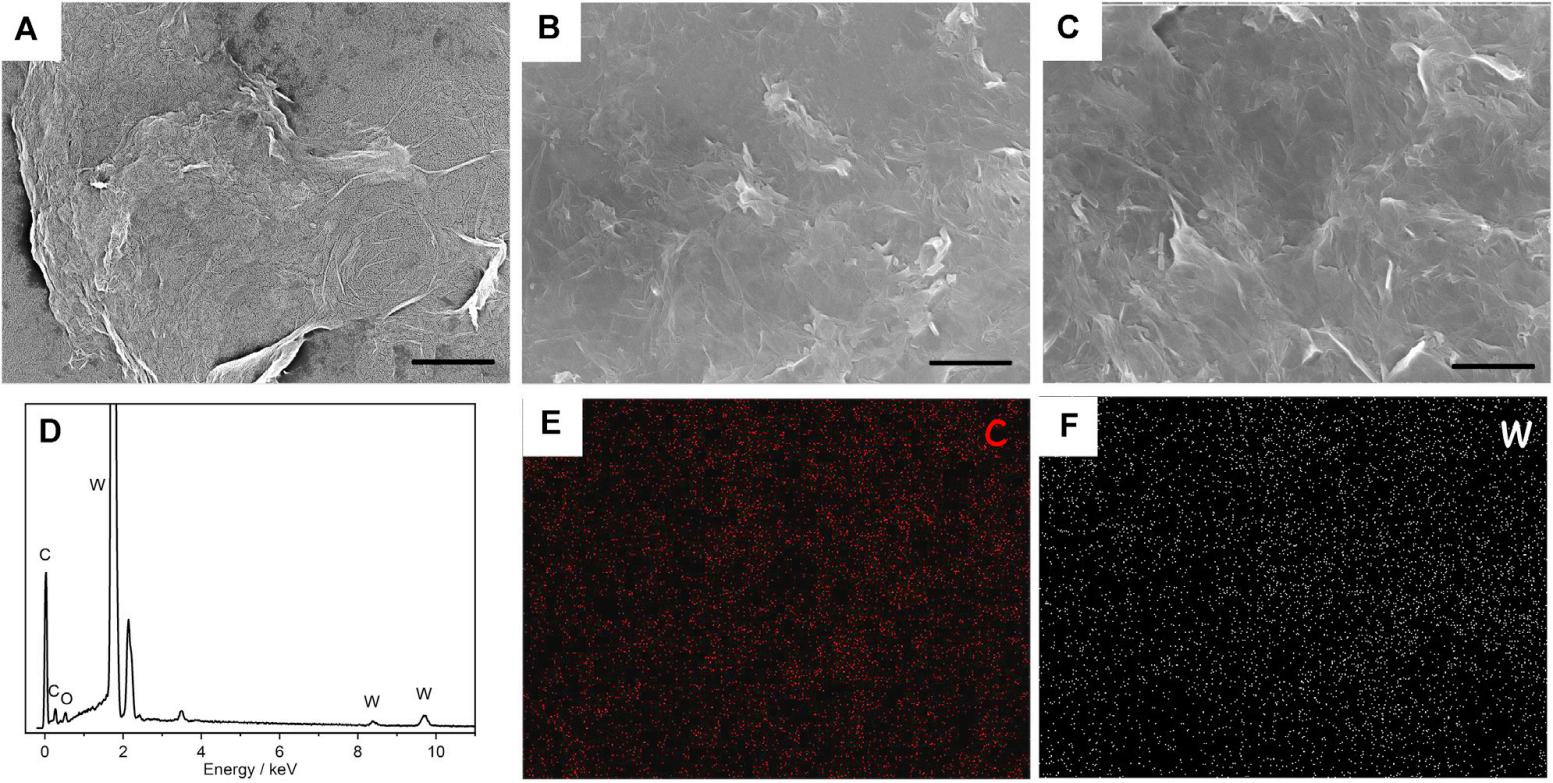
SEM images of (PEI/rGO)_n_-POM composite films with 1 **(A)**, 3 **(B)**, and 5 **(C)** layers. The scale is 2 μm. **(D)** EDX analysis of (PEI/rGO)_3_-POM composite films. Corresponding elemental mapping of C **(E)** and W **(F)** for (PEI/rGO)_3_-POM composite films.

SEM was performed on (PEI/rGO)_n_-POM films to investigate the surface morphology and homogeneity of composite films with different layer numbers, as shown in Figure 2.

A typical SEM image in Figure 2A of (PEI/rGO)_1_-POM shows the GO nanosheet structure mounted on a silicon substrate, which exhibited a crumpled and paper-like structure. Figures 2B, C are (PEI/rGO)_3_-POM and (PEI/rGO)_5_-POM films, respectively. These two films, with different numbers of GO layers, show a similar structure with superposition of the GO nanosheet, indicated as GO multilayer films manufactured by LBL.

*In situ* EDX was used to analyze the composition of (PEI/rGO)_3_-POM films (Figure 2D). The peaks for C and W correspond to rGO and POM attracted in the rGO. The elemental mapping of (PEI/rGO)_3_-POM films shows that the C and W elements (Figures 2E, F) are uniformly distributed on the surface of the films. The result indicates that POM uniformly adsorbed on the rGO surface.

**FIGURE 3:**
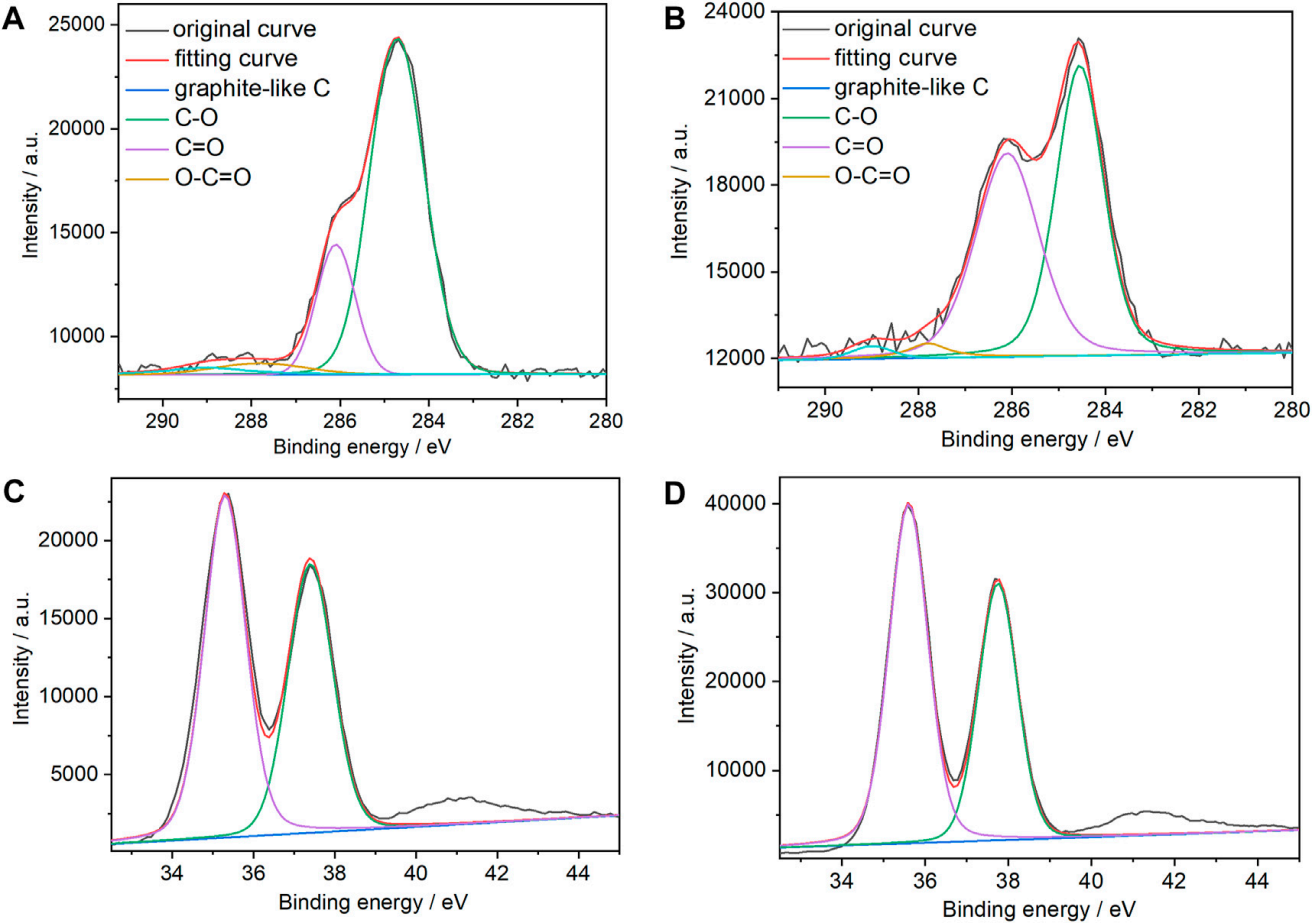
C1s XPS spectra of **(A)** (PEI/rGO)_3_-POM and **(B)** (PEI/GO)_3_/PEI/POM composite films. XPS spectra of W 4f in **(C)** (PEI/rGO)_3_-POM and **(D)** (PEI/GO)_3_/PEI/POM composite films.

XPS spectroscopy was used to verify the composition of the two types of films and the chemical states of the graphene C atoms. Figures 3A, B show the C 1s XPS spectra of the (PEI/rGO)_3_-POM and (PEI/GO)_3_/PEI/POM films. There are four evident types of carbon for (PEI/GO)_3_/PEI/POM films, appearing at 284.7, 286.2, 287.8, and 289.0 eV attributed to different chemical states of GO, which are similar to graphite C, C–O, C=O, and O–C=O (Li et al., 2010), respectively. However, compared to (PEI/GO)_3_/PEI/POM films, the C–O group content in the (PEI/rGO)_3_-POM films decreases from 39.8% to 19.3%. The result indicates that the reduction of POM heterpoly blue could effectively eliminate the oxygen-containing GO groups. Moreover, the graphite-like C group content increased from 50.7% to 73.2%, verifying the restored sp^3^/sp^2^-hybridized carbon structures in the (PEI/rGO)_3_-POM films. The presence of tungsten in the POM was also determined by XPS of the (PEI/rGO)_3_-POM and (PEI/GO)_3_/PEI/POM films (Figures 3C, D). Peaks for W4f 5/2 and W4f 7/2 with binding energies of 37.95 and 35.7 eV were observed in the two types of films. The values for the W peaks indicate a fully oxidized form of tungsten (W^VI^) in POM in films not fabricated by LBL or *in situ* assisted electroreduction.

In addition, comparing the intensity of the C XPS spectra of the (PEI/rGO)_3_-POM and (PEI/GO)_3_/PEI/POM films, the area of the C peaks is almost the same in the two types of films, which is due to the identical manufacturing process used for (PEI/GO)_3_ films, resulting in the same GO content. However, the POM content in (PEI/rGO)_3_-POM films is only approximately 57% of that in (PEI/GO)_3_/PEI/POM films upon comparison of the area of the W peaks in the two types of films. This result is consistent with the results of the UV-Vis absorption spectra.

To compare the permeability of POM to (PEI/GO)_n_ films, XPS spectroscopy was conducted on (PEI/rGO)_n_-POM films with one to six PEI/rGO layers, the results of which are are shown in Supplementary Figure S1. The contents of the graphite-like C group in (PEI/rGO)_n_-POM films with 1 (76.5%) to 2 (75.3%) PEI/rGO layers are similar, and the content in (PEI/rGO)_3_-POM films slightly decreased (73.2%) (Supplementary Table S1). This suggests that POM could penetrate into (PEI/GO)_n_ films with 2.6 layers and reduce GO to rGO at the same time. However, the graphite-like C group content of (PEI/rGO)_4_-POM (69.6%), (PEI/rGO)_5_-POM (65.9%), and (PEI/rGO)_6_-POM (62.1%) films decreased gradually, which is consistent with the extent of POM reduction of (PEI/GO)_n_ films, implying that POM could not penetrate into (PEI/GO)_n_ films with more than three layers. Furthermore, the extent of rGO reduction by POM may affect the catalytic efficiency of (PEI/rGO)_n_-POM films.

According to the aforementioned discussion, there are four interaction forces on (PEI/rGO)_n_-POM films in the manufacturing process. In the LBL assembly process, a cationic polyelectrolyte PEI is adsorbed onto the ITO substrate by electrostatic interaction. After that, GO with a negative charge is bonded by PEI layers with the electrostatic interaction as a driving force. In the electroreduced *in situ* procedure, the electroreduced POM transfers electrons to GO in (PEI/GO)_n_ films, while the POM is adsorbed on rGO as an anionic stabilizer. At the same time, since part of the POM can penetrate into (PEI/GO)_n_ films, a small amount of POM can be adsorbed onto PEI by electrostatic interaction.

### 3.3 Electrochemical and electrocatalytic properties

The CV pattern of (PEI/rGO)_1_-POM films modified with one layer of rGO and POM exhibits four redox waves (I/I′, II/II’, III/III’, and IV/IV’), which indicates a six-electron redox process for W (W^V^/W^VI^) (see inset of Supplementary Figure S2). (PEI/rGO)_n_-POM films with one POM layer and different numbers of PEI/rGO layers were also monitored by CV (Supplementary Figure S2). The area of the enclosed cyclic voltammogram increases as the number of PEI/rGO layers increases. According to the capacitance formula *C=S/2v∆U* (S is the area of the enclosed cyclic voltammogram, v is the scanning speed, and ∆U is the voltage difference), the greater the area of the enclosed curve, the greater the capacitance of the corresponding films (Xiang et al., 2012). It is evident that the capacitance of (PEI/rGO)_n_-POM films increases as the number of PEI/rGO layers increases.

**FIGURE 4:**
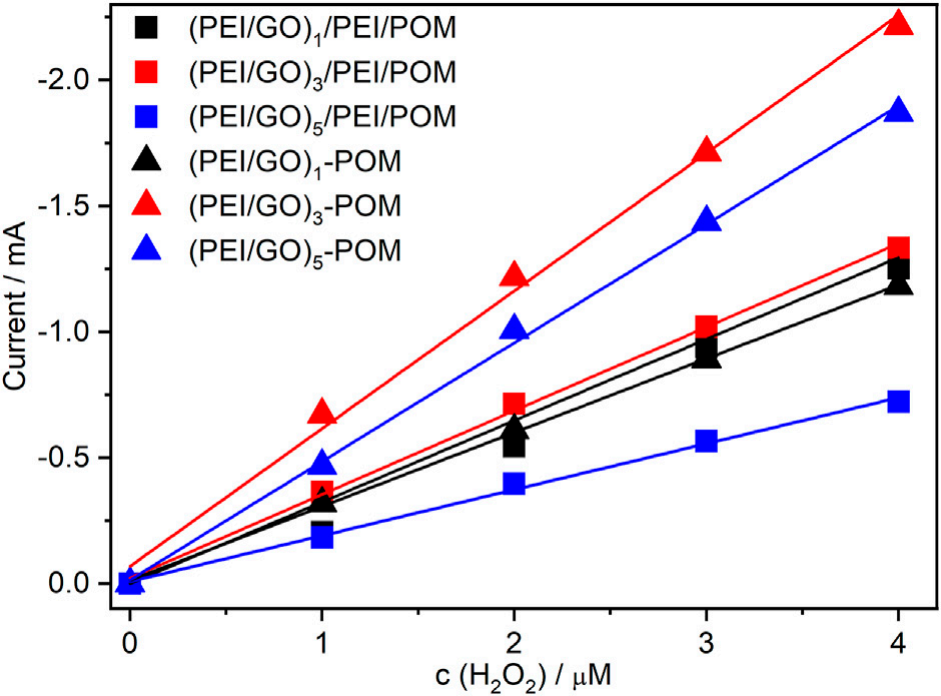
Linear correlation plots of peak currents of (PEI/GO)_n_/PEI/POM and (PEI/rGO)_n_-POM films with different layers vs. the concentration of H_2_O_2_.

According to the literature (Lu et al., 2013; Suo et al., 2016), polyoxometalate nanohybrids have catalytic activity against H_2_O_2_. Therefore, electrocatalytic exploration in H_2_O_2_ was selected as a preliminary test of the catalytic behaviors of prepared films. To compare the catalytic activity for H_2_O_2_ of different types and film layer numbers, cyclic voltammetry was performed. Supplementary Figure S3 shows the CVs of the manufactured (PEI/rGO)_n_-POM films with one (Supplementary Figure S3D), three (Supplementary Figure S3F), and five (Supplementary Figure S3H) GO layers for different H_2_O_2_ concentrations. Evidently, all CVs of (PEI/rGO)_n_-POM films exhibit an increase in W-centered reduction currents with increasing H_2_O_2_ concentrations, while the oxidation peak decreases. The CVs of the (PEI/GO)_n_/PEI/POM films with one (Supplementary Figure S3A), three (Supplementary Figure S3B), and five (Supplementary Figure S3C) GO layers also exhibit increasing reduction currents and decreasing oxidation currents under the aforementioned conditions. Apparently, composite films produced by *in situ* assisted electroreduction can effectively electrocatalyze H_2_O_2_ reduction. Furthermore, comparative investigations of the electrocatalyzed response to H_2_O_2_ reduction, based on cathodic reduction of the six types of films, were used. For convenience, cathode currents at −0.8 V were selected as the catalytic current for each film. All catalytic currents of the composite films exhibit a linear response to H_2_O_2_ concentrations (Figure 4). The linear relationship of different composite films was normalized for comparison. The following points can be obtained: the catalytic efficiency of the two films is similar when *n* = 1 (black curves in Figure 4), while the efficiency of the (PEI/rGO)_n_-POM films is significantly higher than that of (PEI/GO)_n_/PEI/POM films when *n* = 3 or 5 (red and blue curves in Figure 4). However, the POM content in (PEI/rGO)_n_-POM was only approximately half of that in (PEI/GO)_n_/PEI/POM films according to the UV-Vis results (Figure 1). Interesting findings illustrate that the catalytic efficiency of composite films prepared by *in situ* assisted electroreduction is much higher than that of films prepared by the LBL method. This result may be due to electron exchange and adsorption between POM and GO during manufacture of the (PEI/rGO)_n_-POM films. Therefore, POM can directly exchange electrons with PEI/rGO layers in the catalysis process. Furthermore, the GO layers were converted to rGO by reduced heteropoly blue in the reduction process. Structural defects in sp2 carbons caused by oxygen-containing groups of GO were recovered, making the properties of rGO closer to those of graphene than those of GO (Gómez-Navarro et al., 2010; Moon et al., 2010), thus promoting excellent electron transport efficiency in (PEI/rGO)_n_-POM films. However, POM was adsorbed onto GO by PEI on (PEI/GO)_n_/PEI/POM films. In the catalysis process, the POM needs to transfer electrons to the PEI medium before transferring the electrons to the GO layers. The (PEI/rGO)_n_-POM films prepared by *in situ* assisted electroreduction can greatly improve the electron transport efficiency compared to (PEI/GO)_n_/PEI/POM films prepared by LBL. Thus, the current response of (PEI/rGO)_n_-POM films to H_2_O_2_ has been greatly enhanced (Li et al., 2013).

Finally, an electrochemical mechanism for H_2_O_2_ catalyzed on (PEI/rGO)_n_-POM films was proposed, as shown in the following formula (Meng et al., 2011).

$$H_{2}O_{2}+e^{-}\;\to \;{OH}_{ad}+{OH}^{-}$$

$${OH}_{ad}+e^{-}\;\to \;{OH}^{-}$$

$$2{OH}^{-}+2H^{+}\;\to \;2H_{2}O$$

The catalytic efficiency of (PEI/rGO)_n_-POM films with 1 to 6 GO layers was also investigated. The linear relationship of different H_2_O_2_ concentrations catalyzed on (PEI/rGO)_n_-POM films was normalized (Supplementary Figure S4). The slope of the linear relationship first increases and then decreases as the number of PEI/rGO n-layers increases, and reaches a maximum when *n* = 3. The result revealed that increasing the number of PEI/rGO layers results in two opposing factors affecting the catalytic efficiency of (PEI/rGO)_n_-POM films. According to the XPS results, POM can penetrate into 2.6 layers of (PEI/GO)_n_ films and reduce GO to rGO. In view of the high active surface area (Zhang et al., 2015) and high conductivity (Bolotin et al., 2008; Mak et al., 2008) of graphene, the electron transport properties of composite films should improve as the number of PEI/rGO layers is increased; accordingly, electrocatalyzed reduction of H_2_O_2_ is promoted. Therefore, increasing the number of layers to more than three will lead to reduced catalytic efficiency due to the remaining GO not being reduced and adsorbed by the POM (all remaining GO is reduced to rGO by electrodes during the pre-scan procedure). The remaining PEI/rGO layers without POM adsorption cannot promote the electron transport of the redox processes and reduce the catalytic activity due to an increase in layer thickness (Liu et al., 2011b). As a result of both of these factors, the catalytic efficiency is highest when the PEI/rGO layer number is three.

For the (PEI/GO)_n_/PEI/POM films (see Figure 4), the catalytic efficiency increased only slightly when *n* increased from 1 to 3, but greatly decreased when *n* reached 5. According to the UV-Vis results (Figure 1B), the POM catalyst content for (PEI/GO)_n_/PEI/POM films nearly doubled when *n* increased from 1 to 3; however, there is no corresponding doubling of catalytic efficiency but only a small increase. It is implied that the mass transfer resistance caused by the thickness of the GO layers was the main factor in the (PEI/GO)_n_/PEI/POM films. Nevertheless, the catalytic efficiency of (PEI/GO)_n_/PEI/POM films is higher than (PEI/POM)_1_ films that are similar to (PEI/GO)_5_/PEI/POM with the lowest catalytic efficiency (Supplementary Figure S5). The results show that (PEI/GO)_n_/PEI/POM films assembled by the LBL method can improve the catalytic efficiency if the thickness is adequate.

### 3.4 Electrochemical sensor of H_2_O_2_

**FIGURE 5:**
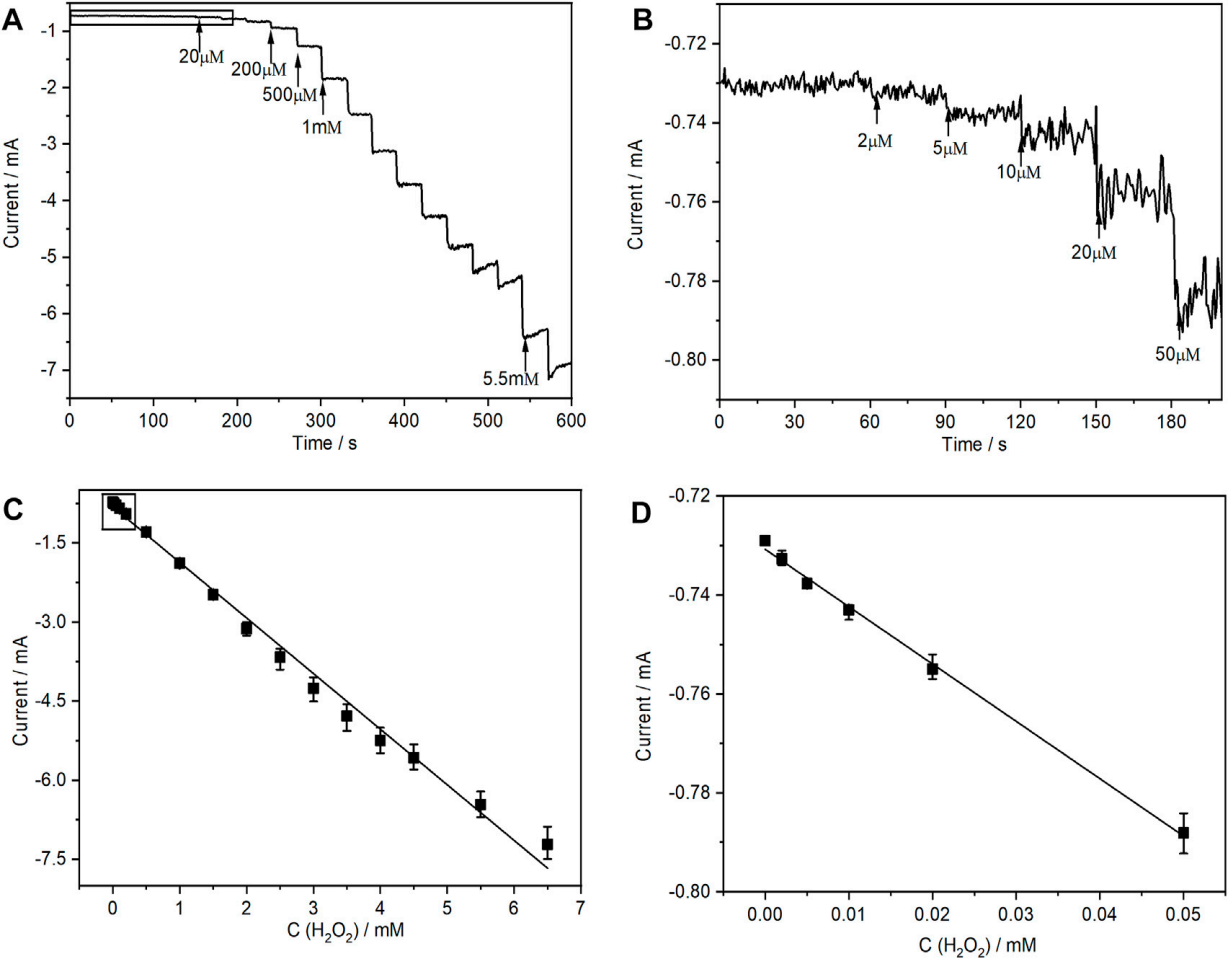
**(A)** Amperometric responses of the (PEI/rGO)_3_-POM films with successive additions of H_2_O_2_ at an applied potential of −0.8 V vs. Ag/AgCl and **(C)** the corresponding calibration plot of steady-state currents vs. the concentration of H_2_O_2_. **(B)** and **(D)** are local parts corresponding to the marked areas in **(A)** and **(B)**, respectively. Error bars represent the standard deviations of three independent experiments.

Amperometric detection was used to verify whether (PEI/rGO)_3_-POM films were a promising type of electrochemical sensor in the H_2_O_2_ test application. The result can provide evaluation of the main characteristics of the sensor. According to the CVs of the (PEI/rGO)_3_-POM films shown in Supplementary Figure S3F, the cathodic reduction peaks disappeared when the H_2_O_2_ concentration increased to 2 mM, due to a significant increase in the catalytic current. For convenience, the applied potential for amperometric detection was selected to be −0.8 V. Figure 5A shows a typical amperometric i-t curve of (PEI/rGO)_3_-POM films after successive addition of different H_2_O_2_ concentrations at 30-s intervals. The applied potential was controlled at −0.8 V for 600 s. With the addition of H_2_O_2_, a well-defined and stable amperometric curve was obtained. The response time was only 1 s after adding a certain amount of H_2_O_2_ (see Figure 5A). Meanwhile, the cathodic current gradually increased as the H_2_O_2_ concentration increased from 2 μM to 6.5 mM. Furthermore, a significant increase in the cathodic current is still be observed when the H_2_O_2_ concentration is as low as 2 μM, which is the lowest value of the linear range (Figure 5B). The calibration curve exhibits a large linear correlation in the range (LRR) from 2.0 × 10^−6^ to 6.5 × 10^−3^ M (Figures 5C, D), while the linear regression equation (LRE) is expressed as I (mA) = −1.05 C_H2O2_(mM) − 0.815 with *R* ^2^ = 0.9930. The limit of detection (LOD) was calculated to be 1.5 μM according to the criterion of S/N = 3. The sensitivity was estimated to be 875 μA mM^−1^·cm^−2^ based on the area of the composite films, which was 1.2 cm^2^.

A comparison of the detection performance of our proposed H_2_O_2_ sensor with previously reported sensors that were prepared as POM- and/or GO-based films is shown in Table 2. All other composite films were modified with catalyst multilayers or Au NPs, while (PEI/rGO)_3_-POM films loaded only the POM monolayer catalyst on the ITO substrate, which is the most economical and effortless method. Compared to other sensors, the proposed sensor has a wider linear range than most others. In particular, the minimum linear range is as low as 2 μM, which is lower than all other sensors. Meanwhile, the detection limit of 1.5 μM is lower than most other reports. Its sensitivity of 875 μA mM^−1^ cm^−2^ is much higher than that of all other sensors and is even approximately 10 times higher than that of K_5_[Ru(bpy)_3_]H_4_PW_18_O_62_/GC (Ammam and Easton, 2012), MWCNTs/[C_8_Py]-PMo_12_/GC (Haghighi et al., 2010), (H_6/5_bppy)_5_[P_2_W_18_O_62_]/graphite (Tian et al., 2010), and Au NPs@POM-GNSs/GC (Liu et al., 2012). The (PEI/rGO)_3_-POM response time is much lower than that of the other sensors in Table 2, which is competent for the fast detection of H_2_O_2_.

**TABLE 2:** Comparison of our as-prepared sensor films and various others for determination of H_2_O_2_.

Electrode	Linear range (μM)	Detection limit (μM)	Sensitivity (μA/mM cm^−2^)	Response time (s)	Reference
(PEI/rGO)_3_-POM/ITO	2–6,500	1.5	875	1	This work
{PEI/(P_8_W_48_/chitosan)_8_}/ITO	25–2,300	1.3	530	5	Lu et al. (2013)
K_5_[Ru(bpy)_3_]H_4_PW_18_O_62_/GC	500–90000	0.5	78	5	Ammam and Easton (2012)
MWCNTs/[C_8_Py]-PMo_12_/GC	20–800	12	81	2	Haghighi et al. (2010)
(H_6/5_bppy)_5_[P_2_W_18_O_62_]/Graphite	20–2,200	13	22.7	4	Tian et al. (2010)
Ti/TiO2/Au/HRP/GC	5–400	2	∼107.9	5	Kafi et al. (2009)
(PDDA/Au@P_5_W_30_/PDDA/GO)_8_/ITO	400–3,200	35	220.9	>20	Suo et al. (2016)
Au NPs@POM-GNSs/GC	5–18000	1.54	58.87	2.5	Liu et al. (2012)

### 3.5 Selectivity, anti-interference, and stability analysis

Selectivity is an important factor for sensors; therefore, the amperometric method was used to investigate the selectivity of (PEI/rGO)_3_-POM films. As shown in Supplementary Figure S6, with the successive addition of electroactive materials, including 20 μM H_2_O_2_, 1 mM methanol, 1 mM ethanol, 1 mM glucose, 1 mM ascorbic acid, and 50 μM H_2_O_2_, the i-t curve displayed an obvious amperometric response immediately after the addition of 20 μM H_2_O_2_ at 30 s. However, there were no distinct amperometric responses upon the subsequent addition of other interferents, even if their concentrations were 20–50 times that of H_2_O_2_. The subsequent addition of 50 μM H_2_O_2_ at 180 s led to a proportional current change in the presence of interferents, which reveals the excellent selectivity of (PEI/rGO)_3_-POM films to H_2_O_2_. High stability is one of the most important prerequisites of a sensor. The stability of (PEI/rGO)_3_-POM films, which were stored in a humid, enclosed environment and not used, was determined by electroactivity measurements at 7, 14, 21, and 28 days, as shown in Supplementary Figure S7. The films retained 99.6%, 96.7%, 94.3%, and 92.1% of their initial amperometric responses. The result indicated that as an electrochemical sensor, (PEI/rGO)_3_-POM films have long-term storage stability.

## 4 Conclusion

An easy, eco-friendly, and green method using POM as an electrocatalyst to reduce GO LBL films by *in situ* assisted electroreduction was developed to manufacture (PEI/rGO)_n_-POM composite films without the use of high temperature or pressure and without noble metals. In this process, GO films were reduced to POM-attracted rGO composite films. rGO restores the conjugated structure of graphene, which greatly promotes the conductivity and electron transport of the films. In the H_2_O_2_ catalysis process, POM can directly exchange electrons with PEI/rGO layers, which greatly improves the catalytic activity of (PEI/rGO)_n_-POM films, whereas for (PEI/GO)_n_/PEI/POM films manufactured by the LBL assembly method, the POM needs to transfer electrons to the PEI medium before the GO layers. Hence, the *in situ* assisted electroreduction method can significantly improve the electrocatalytic activity of (PEI/rGO)_n_-POM films. (PEI/rGO)_3_-POM films with three PEI/rGO layers exhibit the highest efficiency. (PEI/rGO)_3_-POM films bearing a POM monolayer exhibited improved current response, high sensitivity, broad linear range, low detection limit, and fast response to H_2_O_2_ detection. The excellent electrocatalytic performance of the (PEI/rGO)_3_-POM films indicated the competitiveness of nanohybrid sensors in H_2_O_2_ detection. Furthermore, the *in situ* assisted electrochemical methodology can be treated as a general approach to explore other rGO-POM nanohybrid films for potential catalytic activities.

## 5 Supporting information

C1s XPS spectra in (PEI/rGO)_1_-POM (PEI/rGO)_2_-POM, (PEI/rGO)_3_-POM (PEI/rGO)_4_-POM, (PEI/rGO)_5_-POM, and (PEI/rGO)_6_-POM composite films (Supplementary Figure S1); fitting of the C 1s peak binding energy of (PEI/rGO)_n_-POM films with n ranging from 1 to 6 (Supplementary Table S1); cyclic voltammograms of the (PEI/rGO)_n_-POM films with different layers (Supplementary Figure S2); cyclic voltammograms of the (PEI/GO)_n_/PEI/POM films with one, three, and five layers, and the (PEI/rGO)_n_-POM films with 1–6 layers (Supplementary Figure S3); linear correlation plots of peak currents of (PEI/rGO)_n_-POM films with different layers vs. the concentration of H_2_O_2_ (Supplementary Figure S4); linear correlation plots of peak currents of (PEI/GO)_n_/PEI/POM films with different layers and (PEI/POM)_1_ films vs. the concentration of H_2_O_2_ (Supplementary Figure S5) and amperometric responses of the (PEI/rGO)_3_-POM films with successive additions of 20 μM H_2_O_2_, 1 mM methanol, 1 mM ethanol, 1 mM glucose, 1 mM ascorbic acid, and 50 μM H_2_O_2_ at an applied potential of −0.8 V vs. Ag/AgCl (Supplementary Figure S6). Amperometric response of the (PEI/rGO)_3_-POM film to 1 mM H_2_O_2_ at an applied potential of −0.8 V vs. Ag/AgCl at different days (Supplementary Figure S7).

## Data Availability

The original contributions presented in the study are included in the article/Supplementary Material; further inquiries can be directed to the corresponding author.
