# Supplementary material for: Co-assembly of graphene/polyoxometalate films for highly electrocatalytic and sensing hydroperoxide
Source: Front Chem. 2023 May 18;11:1199135. doi: 10.3389/fchem.2023.1199135 (PMC10233151; doi:10.3389/fchem.2023.1199135)
Supplement: Supplementary file 1 [file DataSheet1.doc]

**Supplementary material**

**Co-assembly of graphene/polyoxometalate films for highly electrocatalytic and sensing hydroperoxide**

Yayan Bao ^a^,^b^, Zezhong Chen ^b^, Yuzhen Wang ^b^, Lizhen Liu ^b^, Haiyan Wang ^b^, Zuopeng Li ^b^, Feng Feng ^a,b^*

^a^ School of Chemistry and material science, Shanxi Normal University, Linfen 041004, P. R. China

^b^ College of Chemistry and environmental engineering, Shanxi Datong University, Datong 037009, P. R. China

E-mail: [feng-feng64@263. net](../../../../H:%5Cpaper%5Cfeng-feng64@263.net); Tel.: +86 352 7158662; Fax: +86 352 6100028

Number of Pages: 5
Number of Figures: 6
Number of Table: 1

contents

[**Figure S1 2**](#__RefHeading___Toc61972382)

[Table S1 3](#__RefHeading___Toc61972383)

[Figure S2. 3](#__RefHeading___Toc61972384)

[Figure S3. 4](#__RefHeading___Toc61972385)

[Figure S4. 4](#__RefHeading___Toc61972386)

[Figure S5. 5](#__RefHeading___Toc61972387)

[Figure S6. 5](#__RefHeading___Toc61972387)


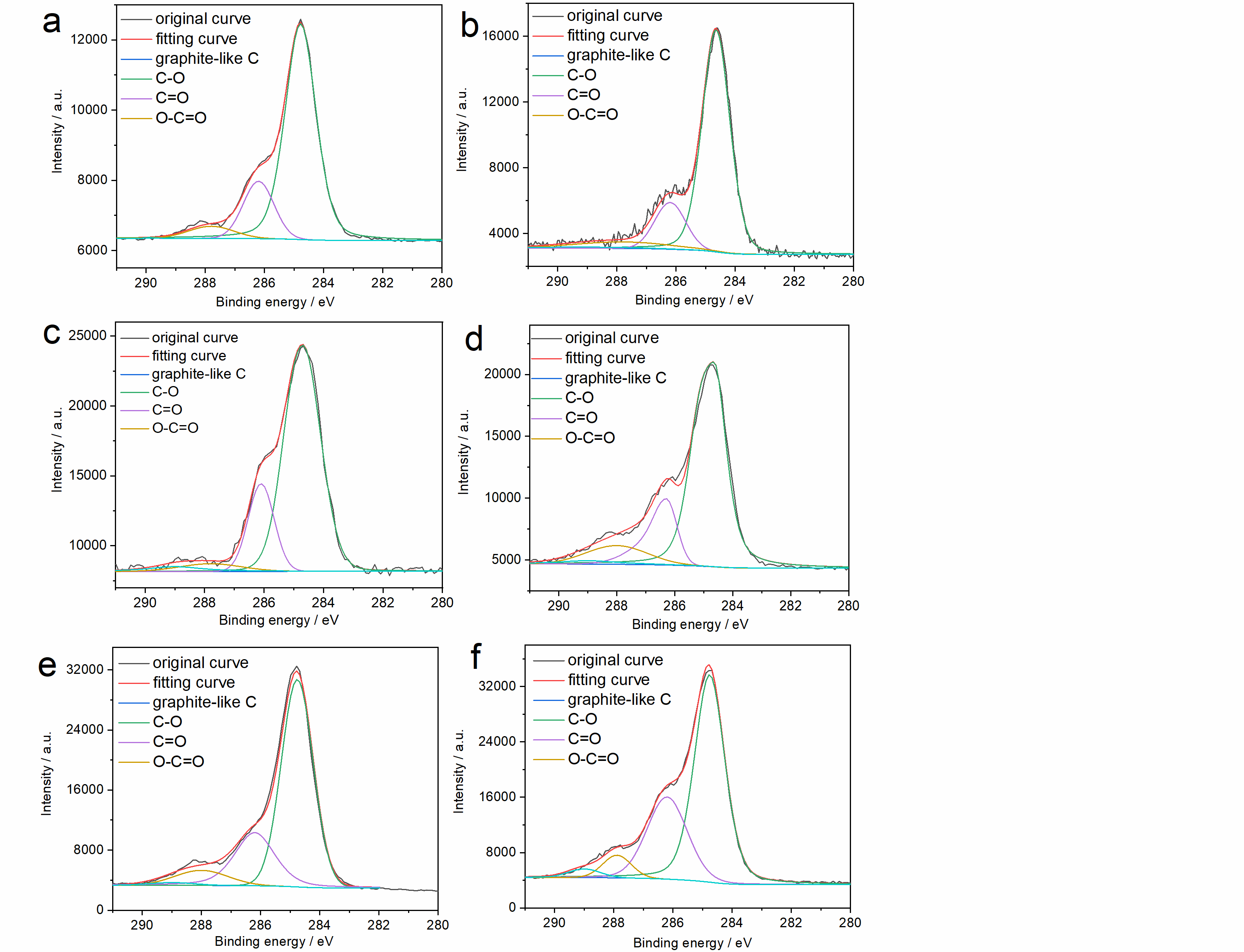


**Figure S1.** C1s XPS spectra in (a) (PEI/rGO)_1_-POM, (b) (PEI/rGO)_2_-POM, (c) (PEI/rGO)_3_-POM, (d) (PEI/rGO)_4_-POM, (e) (PEI/rGO)_5_-POM and (f) (PEI/rGO)_6_-POM composite films.

**Table S1.** Fitting of the C 1s peak binding energy (eV) (relative atomic percentage %) of (PEI/rGO)_n_-POM films with n from 1 to 6.

| Composite films | graphite-like C  (284.7) | C-O  (286.2) | C=O  (287.8) | O-C=O  (289) |
| --- | --- | --- | --- | --- |
| (PEI/rGO)_1_-POM | 76.5 | 17.9 | 5.6 | 0 |
| (PEI/rGO)_2_-POM | 75.3 | 16.2 | 7.1 | 1.4 |
| (PEI/rGO)_3_-POM | 73.2 | 19.3 | 4.2 | 3.3 |
| (PEI/rGO)_4_-POM | 69.6 | 19.7 | 8.5 | 2.2 |
| (PEI/rGO)_5_-POM | 65.9 | 25.0 | 7.9 | 1.2 |
| (PEI/rGO)_6_-POM | 62.1 | 30.0 | 5.6 | 2.4 |

**
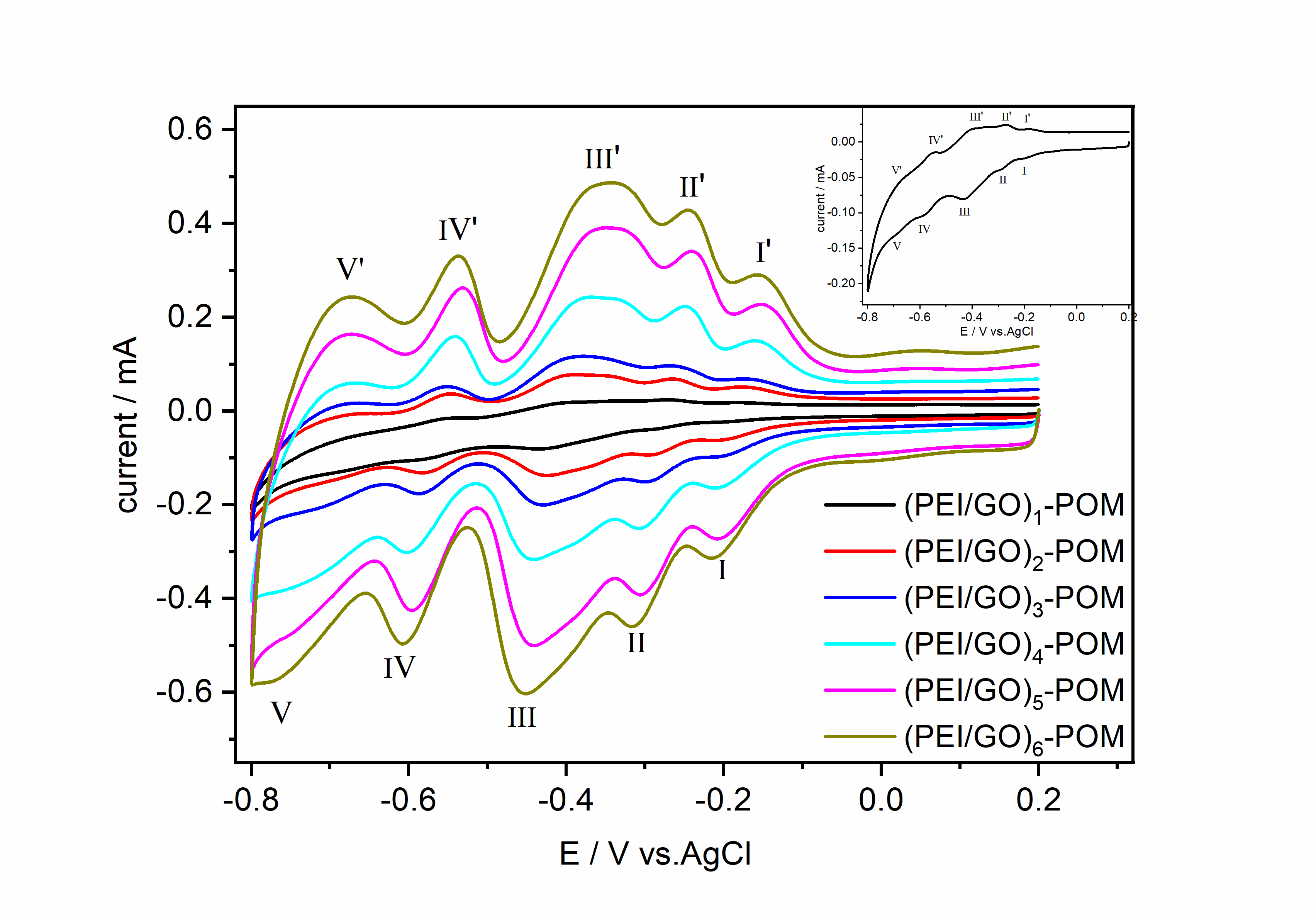
**

Figure S2. Cyclic voltammograms of the (PEI/rGO)_n_-POM films with different layers at 50 mV·S^-1^. The electrolyte is 0.5 M H_2_SO_4_-Na_2_SO_4_ buffer solution with pH = 2.5. Inset: the detail of (PEI/rGO)_1_-POM film.


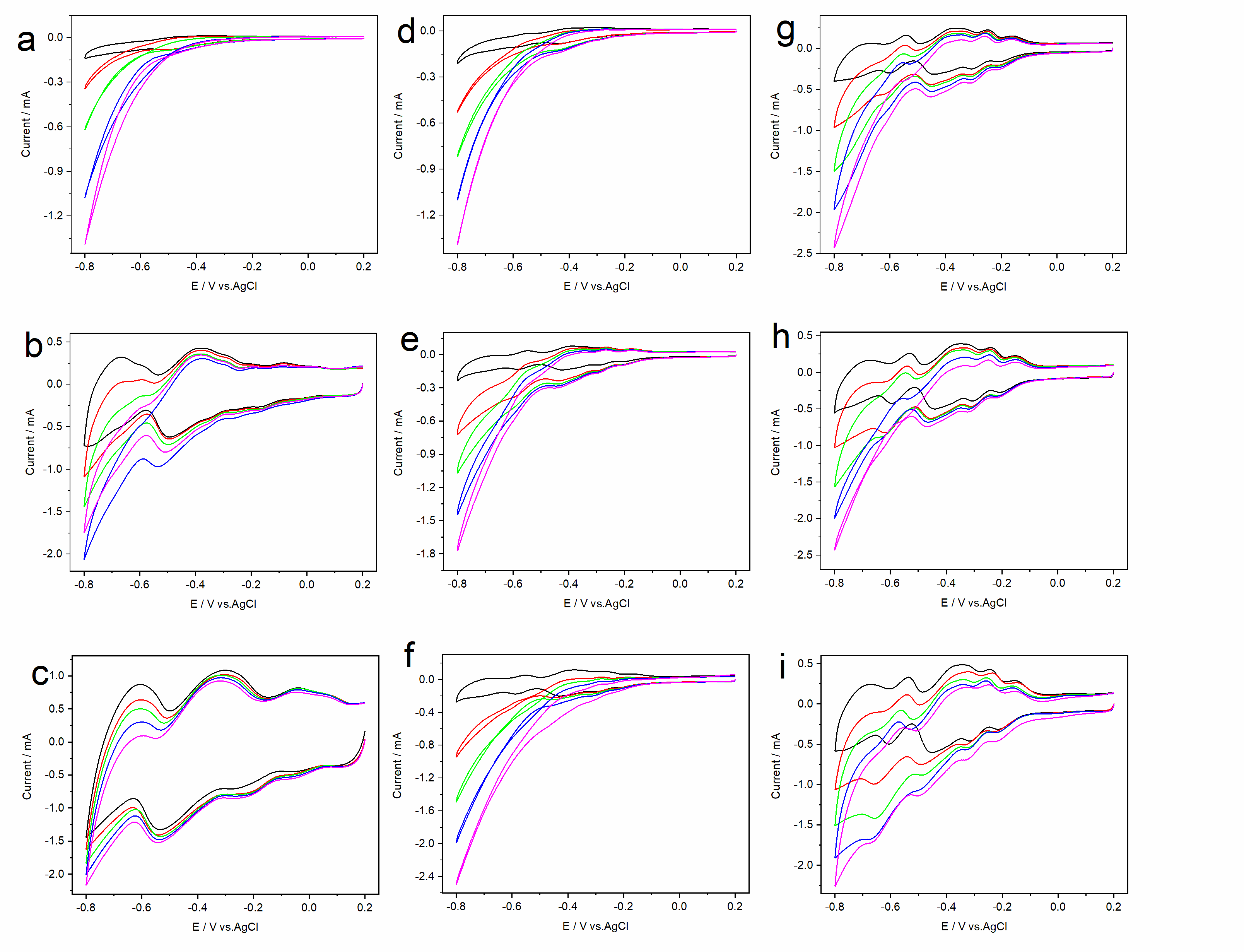


Figure S3. Cyclic voltammograms of the (PEI/GO)_n_/PEI/POM films with 1(a), 3(b) and 5(c) layers, as well as the (PEI/rGO)_n_-POM films with 1(d), 2(e), 3(f), 4(g), 5(h) and 6(i) layers at 50 mV·S^-1^ in the presence of H_2_O_2_ with various concentrations of 0, 1, 2, 3 and 4 mM. The electrolyte is 0.5 M H_2_SO_4_-Na_2_SO_4_ buffer solution with pH = 2.5.


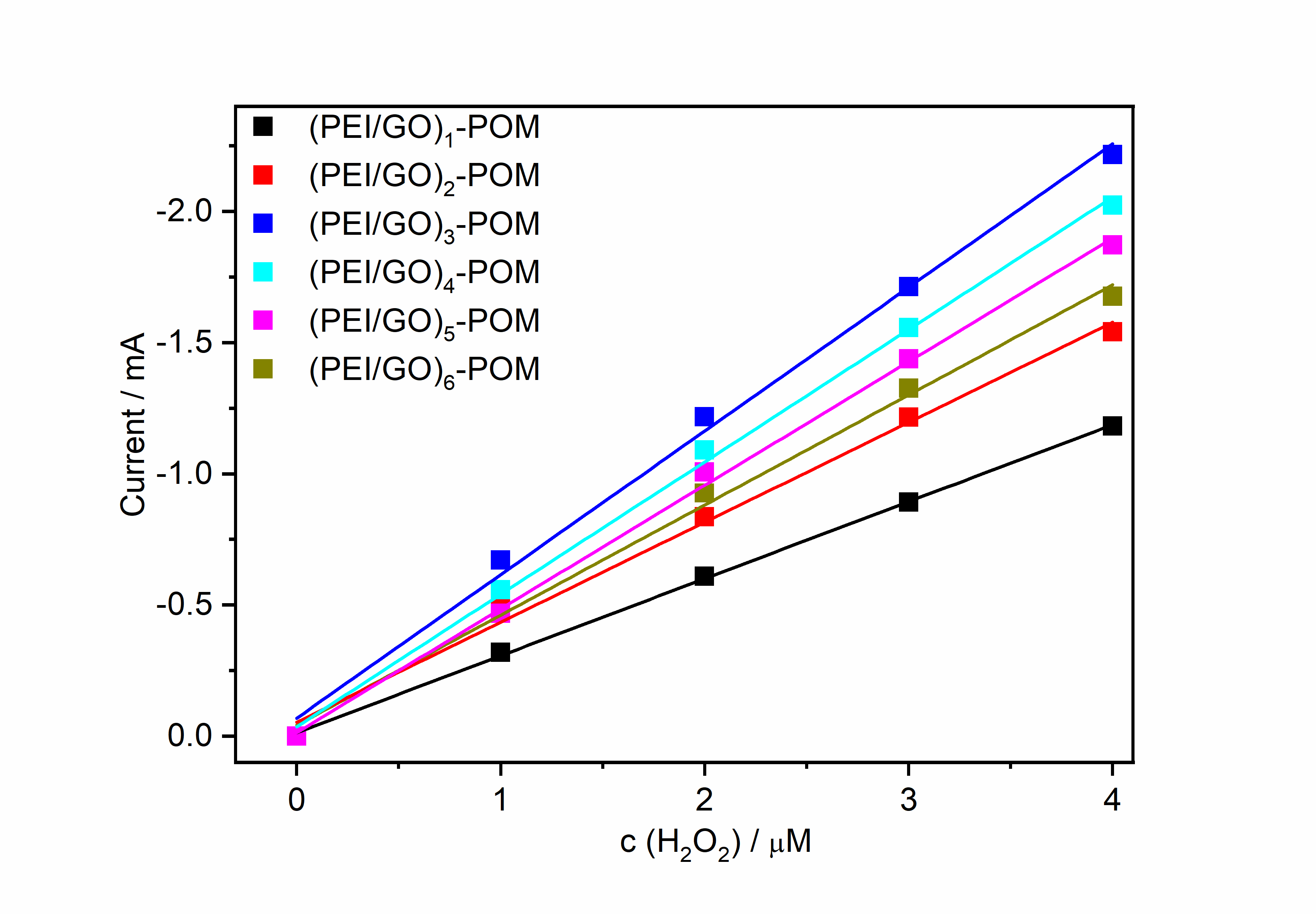


Figure S4. The linear correlation plots of peak currents of (PEI/rGO)_n_-POM films with different layers *vs.* the concentration of H_2_O_2_.


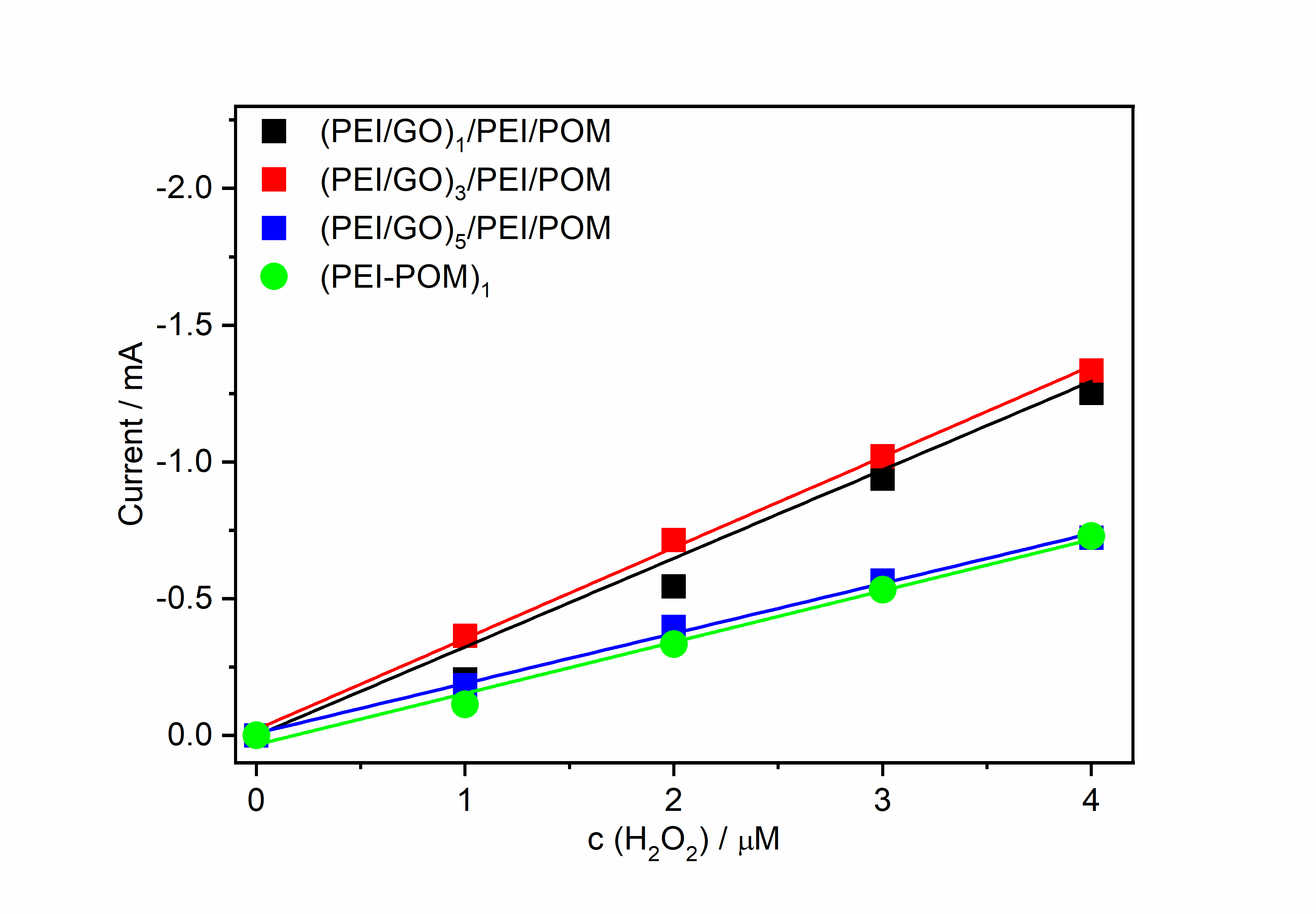


Figure S5. The linear correlation plots of peak currents of (PEI/GO)_n_/PEI/POM films with different layers and (PEI/POM)_1_ films *vs.* the concentration of H_2_O_2_.


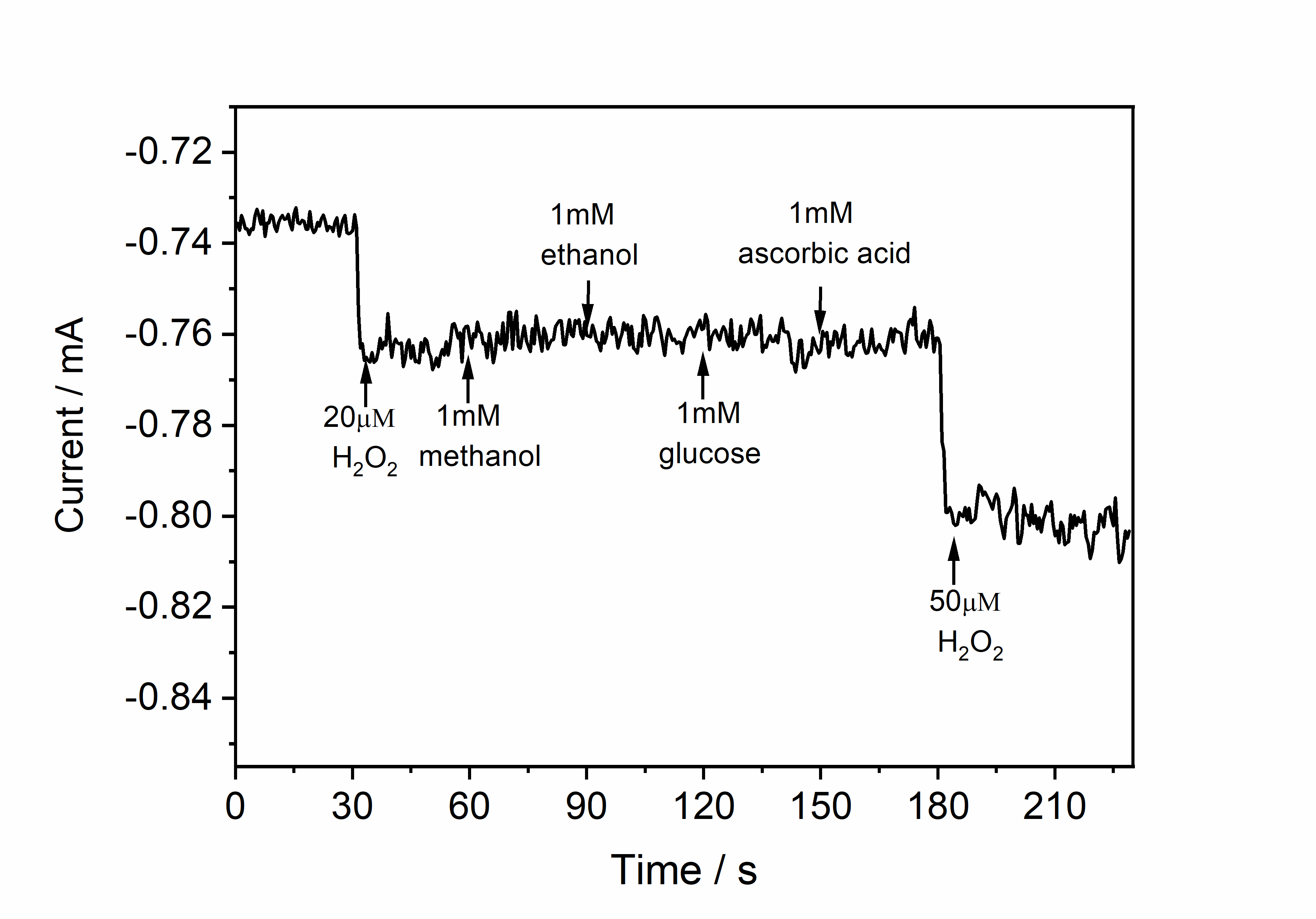


Figure S6. Amperometric responses of the (PEI/rGO)_3_-POM film with successive additions of 20μM H_2_O_2_, 1mM methanol, 1mM ethanol, 1mM glucose, 1mM ascorbic acid, and 50μM H_2_O_2_ at applied potential of -0.8 V *vs.* Ag/AgCl.

**
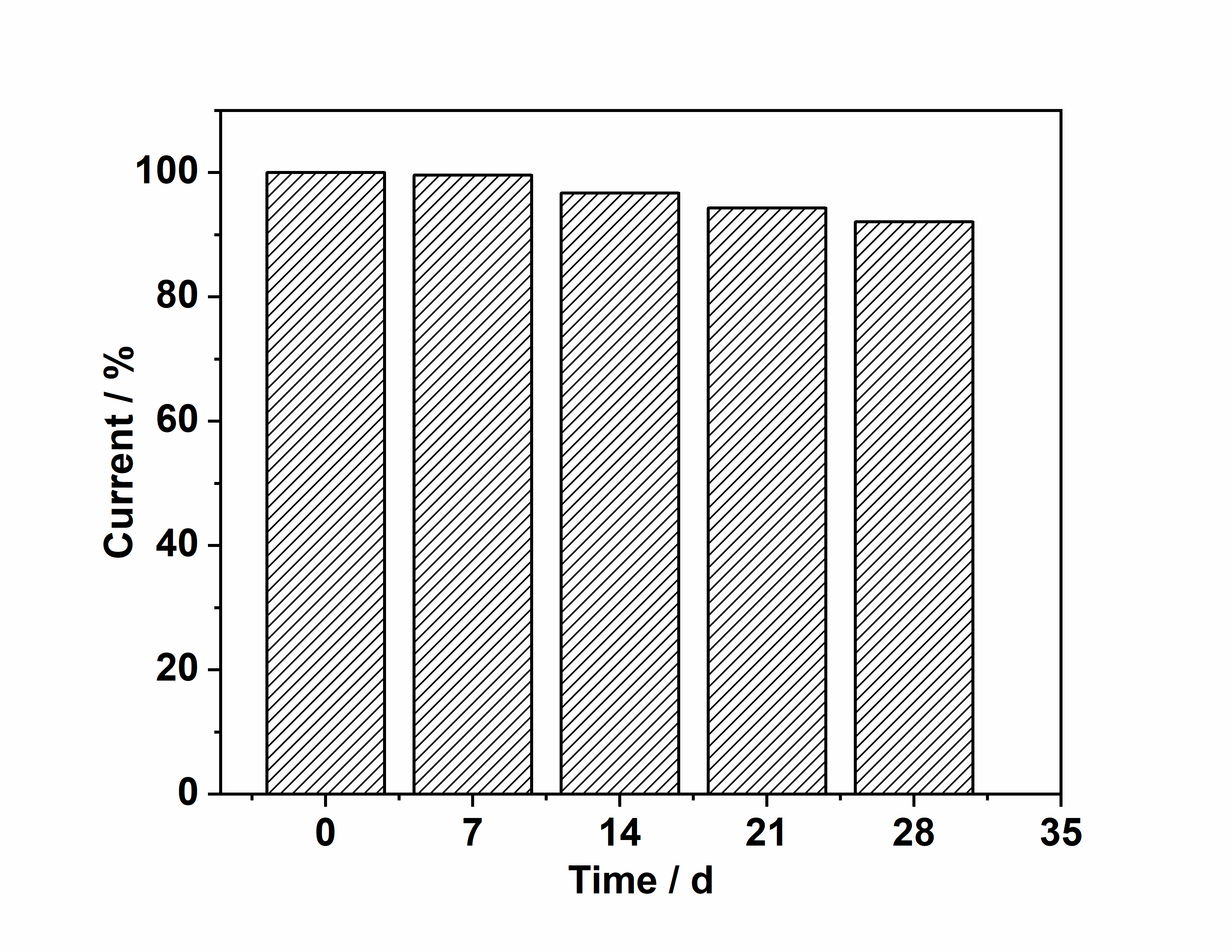
**

Figure S7. The amperometric responsed of the (PEI/rGO)_3_-POM film to 1 mM H_2_O_2_ at applied potential of -0.8 V *vs.* Ag/AgCl at different days.
